# Rheology Modulation and Melt Strength Enhancement Enable Continuous Melt Spinning of PEK-C Fibers

**DOI:** 10.3390/polym18172106

**Published:** 2026-08-30

**Authors:** Yi Mao, Zhao Ke, Ying Ma, Hui Li, Zexu Hu

**Affiliations:** 1 State Key Laboratory of Advanced Fiber Materials, Center for Advanced Low-Dimension Materials, College of Materials Science and Engineering, Donghua University, Shanghai 201620, China; 2230375@mail.dhu.edu.cn (Y.M.); 1239750@mail.dhu.edu.cn (Z.K.); 2 Key Laboratory of Textiles Science and Technology of Ministry of Education, College of Textiles, Donghua University, Shanghai 201620, China; yingma@dhu.edu.cn; 3 College of Mechanical Engineering, Donghua University, Shanghai 201620, China

**Keywords:** fibers, PEK-C, melt spinning, rheological properties, melt strength

## Abstract

Phenolphthalein poly (aryl ether ketone) (PEK-C) is highly compatible with epoxy resin, which is applied in epoxy resin-based composites to improve their strength. Solution spinning and electrospinning techniques are often used to produce PEK-C fibers, but these techniques pollute the environment and are not productive. To address the above challenges, melt spinning may be employed, but due to the high degree of molecular chain entanglement and poor melt fluidity of PEK-C, melt spinning remains difficult. Herein, this study presents the continuous melt spinning of PEK-C fibers through rheology modulation and melt strength enhancement. A series of PEK-C samples with different molecular weights were prepared by adjusting the molar ratio of monomers. The influence of molecular weights on rheological properties was investigated via capillary rheology tests. PEK-C with molecular weight (2.01 × 10^4^ g/mol) was chosen, and the optimal spinning temperature was determined to be 370 °C. The as-polymerized PEK-C powder was compounded into pellets; subsequently, the PEK-C pellets with enhanced melt strength were continuously processed into fiber through melt spinning. The obtained PEK-C fibers possess high tensile strength (0.56 cN/dtex) and breaking strength (1.06 cN/dtex). The mechanical properties and molecular chain orientation of PEK-C fibers were also optimized by spinning process parameters. This work presents continuous melt spinning of PEK-C fibers via rheology modulation and melt strength enhancement, which resolve the environmental and low productivity issues.

## 1. Introduction

Epoxy resin-based composites have been utilized extensively in the aerospace industry, automotive manufacturing and other areas of engineering due to their excellent properties, such as high strength, excellent thermal resistance and dimensional stability [1,2,3]. However, the highly crosslinked network structure of cured epoxy resins produces brittleness and poor fatigue resistance [4,5], which limits their application in extreme working environments, especially at high impact loads and recurring mechanical loads [6,7]. Enhancing the toughness of epoxy resin systems without decreasing their mechanical and thermal properties has, therefore, emerged as an important research topic [8,9,10].

Incorporating high-performance thermoplastic polymer fibers as the reinforcing phase is an effective method for enhancing the toughness of epoxy resin-based composites [11,12,13]. Among various thermoplastic polymers, phenolphthalein poly (aryl ether ketone) (PEK-C) has become of considerable interest due to its specific molecular structure, that is, aromatic rings, ether linkages, and ketone groups [14,15]. PEK-C has excellent mechanical strength, thermal stability and chemical resistance [16,17]. Moreover, PEK-C is highly compatible with epoxy resins and has been widely used as a toughener in epoxy composites [18,19,20]. A thermoplastic–thermoset continuous phase structure is created when PEK-C and epoxy resin are mixed after curing. The interface between the two phases can prevent crack propagation. In addition, the thermoplastic phase absorbs energy through yielding deformation, which enhances the toughness of epoxy resin [21,22,23]. However, as the loading of PEK-C toughener increases, the solubility and fluidity of epoxy resins significantly decrease, which severely restricts the processability of epoxy resin [24,25]. The interaction and blending of PEK-C with epoxy matrices remain a focal point for toughening mechanisms [26,27,28]. In contrast, incorporating PEK-C fibers into the epoxy matrix can not only increase the mechanical strength and interlaminar toughness of the composites but also maintain structural stability during the molding and processing of the composites. Therefore, PEK-C fibers can serve as high-performance reinforcement materials in advanced composite applications [29,30].

Currently, PEK-C fibers are primarily fabricated via solution spinning and electrospinning techniques. However, both methods are time-consuming and involve extensive use of volatile organic solvents in the process, which pollutes the environment and demands a recovery cost of the solvents with a time-consuming manufacturing process [31,32]. Both methods fail to meet the demands of industrial large-scale fabrication and applications because of the low production efficiency [33,34,35]. In contrast, melt spinning is a solvent-free, environmentally friendly and highly efficient fiber-forming technology that has many benefits for the mass production of the industry [36]. However, no relevant studies have been reported to date, because of a severe challenge with continuing the melt spinning of PEK-C fibers. First, the PEK-C molecule structure consists of aromatic rings and bulky phenolphthalein side groups that result in high molecular chain entanglements, high melt viscosity and poor flow during their melt processing. At the same time, the melt strength is insufficient to ensure stable filament formation during melt spinning, resulting in poor spinnability [37,38].

This study proposes a melt spinning strategy based on the regulation of rheological properties and enhancement of melt strength to achieve the continuous melt spinning of PEK-C fibers. A series of PEK-C samples with gradient molecular weights were synthesized by precisely adjusting the molar ratio of monomers during polymerization. The influence of molecular weight on the rheological properties of PEK-C was systematically investigated through capillary rheology tests. Based on the rheological analysis, PEK-C with molecular weight of 2.01 × 10^4^ g mol^–1^ was selected for melt spinning. The corresponding optimal spinning temperature was determined to be 370 °C. Subsequently, PEK-C powder was processed into pellets and fed into the melt spinning machine, whereby continuous melt spinning PEK-C pre-oriented fibers were obtained. The results demonstrated that the PEK-C fibers had excellent mechanical properties, with a tensile strength of 0.56 cN/dtex and a breaking strength of 1.06 cN/dtex, respectively. Moreover, the spinning process parameters were optimized based on the mechanical properties and molecular chain orientation of PEK-C fibers. This work successfully achieves the continuous melt spinning of PEK-C fibers and provides an important material foundation and technical framework for the development of high-performance, environmentally friendly composites.

## 2. Materials and Methods

### 2.1. Materials

Phenolphthalein (PHT, >99.5%) and the 4,4′-difluorobenzophenone (DFK, >99.5%) were obtained from Shanghai Haohong Biomedical Technology Co., Ltd. (Shanghai, China), Tetramethylene sulfone (TMS, >99.5%) was supplied by Liaoning Guanghua Chemical Co., Ltd. (Liaoyang, China), and dried sodium carbonate (Na_2_CO_3_, >99.5%) and xylene (XYL, >99.5%) were purchased from Shanghai Aladdin Chemicals (Shanghai, China).

### 2.2. Preparation of PEK-C

**Figure 1:**
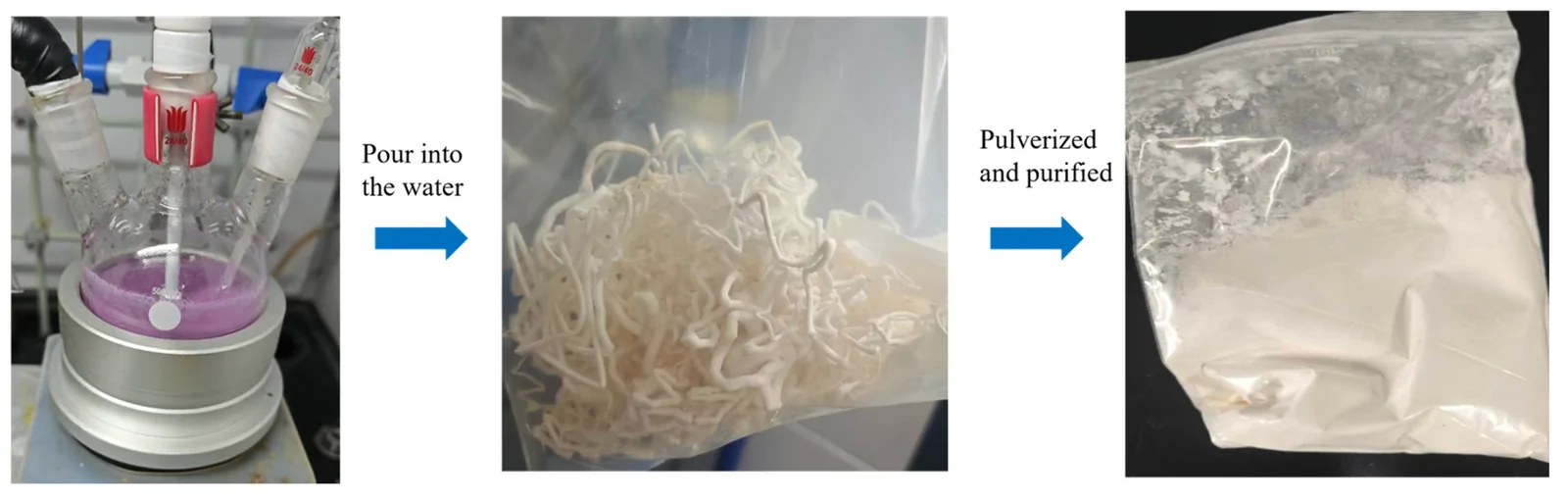
Flowchart of PEK-C polymerization process.

Figure 1 shows a flowchart of PEK-C polymerization process. PEK-C was synthesized via a nucleophilic substitution polycondensation reaction. All polymerization reactions were independently repeated in triplicate to verify process stability. For a representative DFK/PHT molar ratio of 1.044, the reaction was carried out in a three-necked flask, equipped with a mechanical stirrer, a thermometer, and water separation apparatus. Thus, 18.15 g of anhydrous sodium carbonate, 30 g of DFK, 41.92 g of PHT, 30 mL of xylene and 220.5 g of Tetramethylene sulfone were added; the solid content of the resulting solution was 25%. With continuous flowing nitrogen and stirring, the reaction mixture was heated to 185 °C, then refluxed with water separation for 2.5 h. The water separation apparatus was opened and we removed the xylene. Subsequently, the temperature was maintained at 220 °C for 6.5 h to complete the reaction. The final reaction mixture was slowly poured into deionized water, yielding white, strip-shaped products. After soaking in water for 10 h, the products were pulverized. Subsequently, the products were heated and stirred in deionized water for 1 h, then filtered; this process was repeated eight times until the filtrate was clear and colorless. Finally, the purified samples were dried in a forced-air oven at 180 °C for 4 h, yielding white polymer powder.

### 2.3. Melt Spinning of PEK-C

The powder of PEK-C was placed in a vacuum oven and dried at 150 °C under −0.1 MPa for 12 h. Then, a twin-screw extruder was employed for extrusion pelletizing, with the barrel temperatures set to 300 °C (Barrel 1), 350 °C (Barrels 2–3), 360 °C (Barrel 4), 365 °C (Barrel 5), and 370 °C (Barrels 6–7). The die head temperature was maintained at 370 °C. The dried powder was fed into the extruder hopper and was conveyed by the screw. Continuous cylindrical strands were extruded through the die head and transported to the pelletizer. To obtain yellow PEK-C pellets, the toughened strands were sliced into pellets in the shape of cylinders measuring about 3 mm in length and 2 mm in diameter.

The PEK-C pellets were dried in a nitrogen-filled oven for 6 h before melt spinning process. Subsequently, the melt spinning of PEK-C was performed with a homemade melt spinning machine. Temperatures of different parts of the spinning machine were set to 358 °C (the C1 section of screw), 370 °C (the C1–C4 sections of screw), 372 °C (melt tube, spinning pack and pump). To prevent the expensive and delicate spin pack from being clogged or scratched by carbonized particles and foreign impurities, trial extrusion and melt purging were performed without the spin pack installed. After PEK-C pellets were fed into the extrusion system via hopper, the melt was extruded at a low screw speed for material testing and purging. According to the trial extrusion and purging performance of PEK-C samples, PEK-C, with optical molecular weight, was chosen for melt spinning. A circular spinneret with a diameter of 0.50 mm and a length-to-diameter ratio of 5/0.5 was selected for the spinning assembly. The spinneret was configured with 24 orifices, and the screw pressure was maintained at 13 MPa. Under the metered delivery of the metering pump, the PEK-C melt was continuously fed into the spinneret and extruded through the micropores of the spinneret, forming continuous filamentary melt streams. The melt streams subsequently entered a chamber, where rapid heat exchange occurred under crossflow air blowing. The melt temperature rapidly dropped below the glass transition temperature, completing cooling, solidification and preliminary structural setting, and yielding unwound PEK-C as-spun fibers. Following further wind-up collection of the fibers, the winding drum speed was maintained at 600 m/min. By adjusting the rotation speed of the metering pump to 21 and 24 rpm, two types of pre-oriented fibers were obtained.

### 2.4. Characterization

The ^1^H NMR spectra were acquired using a spectrometer (Bruker 600 AVANCE III, Bruker Corporation, Karlsruhe, Germany) with deuterated dimethyl sulfoxide (DMSO-d_6_) as the solvent. The FT-IR spectrum was recorded using a spectrometer (Nicolet 6700, Thermo Scientific Inc., Waltham, MA, USA). The wavenumber range was 4000–400 cm^–1^ and the number of scans was 32; the tests were carried out in ATR mode. The molecular weight and distribution of the polymers were determined using a multi detector GPC/SEC system (1260 Infinity II, Agilent Technologies Co., Ltd., Santa Clara, CA, USA) with tetrahydrofuran (THF) as the solvent. The signals were detected using refractive index detector (Agilent Technologies Co., Ltd., Santa Clara, CA, USA) and evaluated using polystyrene reference calibration. The thermal weight loss behavior of the polymer was characterized by TGA (TGA 550, TA Instruments, New Castle, DE, USA). The tests were performed under a nitrogen atmosphere with a heating rate of 10 °C/min from room temperature up to 800 °C. The real-time weight loss variation with temperature was monitored to analyze the thermal decomposition temperature and high-temperature resistance. The glass transition temperature (Tg) and thermal crosslinking reaction temperature were tested using DSC (Discovery DSC 250, TA Instruments, New Castle, DE, USA). DSC measurements for Tg were conducted in a nitrogen atmosphere with a heating rate of 10 °C/min. The sample weighing 5–10 mg was heated to eliminate thermal history and followed by a cooling and reheating cycle to record Tg during the second heating scan. DSC measurements for thermal crosslinking reaction were performed under a nitrogen atmosphere with a heating rate of 20 °C/min. The sample weighing 5–10 mg was heated to targeted temperature and maintained for 30 min to monitor the thermal crosslink reaction. The melt flowability was measured using MFI tester (FBS-400B, Xiamen Fubusi Testing Equipment Co., Ltd., Xiamen, China) under test conditions of 360 °C with a 5 kg load. The intrinsic viscosity of the polymer was measured using an Ubbelohde viscometer tester (SBQ81834, Shanghai Xingao Instrument Co., Ltd., Shanghai, China) with a capillary inner diameter of 0.5 mm. A 0.5 g/dL polymer solution was prepared using N-methyl-2-pyrrolidone (NMP) as the solvent, and the measurement was conducted at 25 °C. Capillary rheology tests were carried out using a capillary rheometer (Capillary Rheometer RG25, Gottfert Ltd., Buchen, Germany). During the tests, a capillary die with dimensions of 5/0.5 mm was employed. Rheological tests were conducted at 350, 355, 360, 365, and 370 °C, over a shear rate range of 1000–10,000. The flowchart of the PEK-C fiber-forming process was generated with the assistance of Google Gemini 3.1 Pro (Google LLC, Mountain View, CA, USA). The tool was used solely for the visualization and graphical organization of the melt-spinning and subsequent hot-drawing processes. The surface and cross-section micromorphology of PEK-C fibers was characterized using electron microscopy (SEM Regulus 8230, Hitachi, Japan). After gold sputtering for 2 min, the morphologies of PEK-C fibers were observed via SEM (Regulus 8230, Hitachi, Japan) at an accelerating voltage of 15 kV under magnifications of 50×, 200× and 500×. Based on the acquired SEM images, quantitative statistical analysis of fiber diameter and its distribution was performed using ImageJ (Version 1.54g, National Institutes of Health, Bethesda, MD, USA), with no fewer than 20 filaments counted for each sample. The birefringence (Δn) of the fibers was measured using an SSY-KN birefringence tester (Shanghai Kailidi New Material Technology Co., Ltd., Shanghai, China). The instrument was focused until a clear image of the fiber was obtained. The CTB optical compensator was inserted into the optical path, and its compensation angle was adjusted to record angles θ_1_ and θ_2_, which corresponded to the complete extinction states achieved by rotating the compensator in opposite directions. Δn was subsequently calculated from the absolute value of the difference between θ_1_ and θ_2_ and the average fiber diameter. The mechanical properties of the fiber multifilament were tested using a YG029A fully automatic single-yarn strength tester produced by Changzhou Dahua Electronic Instrument Co., Ltd. (Changzhou, China). The test standard is based on the national standard GB/T 14344-2022 test methods for tensile Properties of Chemical Fiber Filaments [39]. All the data were collected from three independent tests.

## 3. Results and Discussion

### 3.1. The Polymerization Reaction of PEK-C

**Figure 2:**
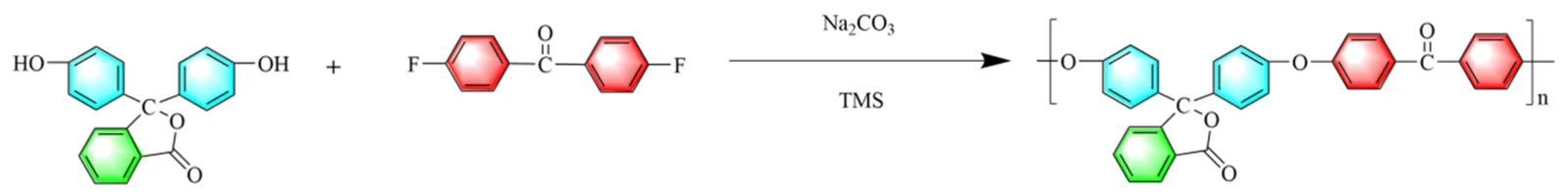
The chemical equation for the PEK-C polymerization reaction. The different colors are used to distinguish the benzene rings: the blue hexagons represent the phenyl rings in PHT; the green hexagons represent the benzene rings in the phenolphthalein side-group; the red hexagons represent the benzene rings in DFK.

In this study, PEK-C was synthesized via nucleophilic substitution polycondensation, with the reaction equation shown in Figure 2. During the polymerization, excess 4, 4′-difluorobenzophenone (DFK) was used for end-capping.

Five PEK-C samples with different molecular weights were prepared by adjusting the molar ratio of DFK to phenolphthalein (PHT), as listed in Table 1. The PEK-C products were sequentially named PEK-C-1, PEK-C-2, PEK-C-3, PEK-C-4, and PEK-C-5 in ascending order of the molar ratio.

**Figure 3:**
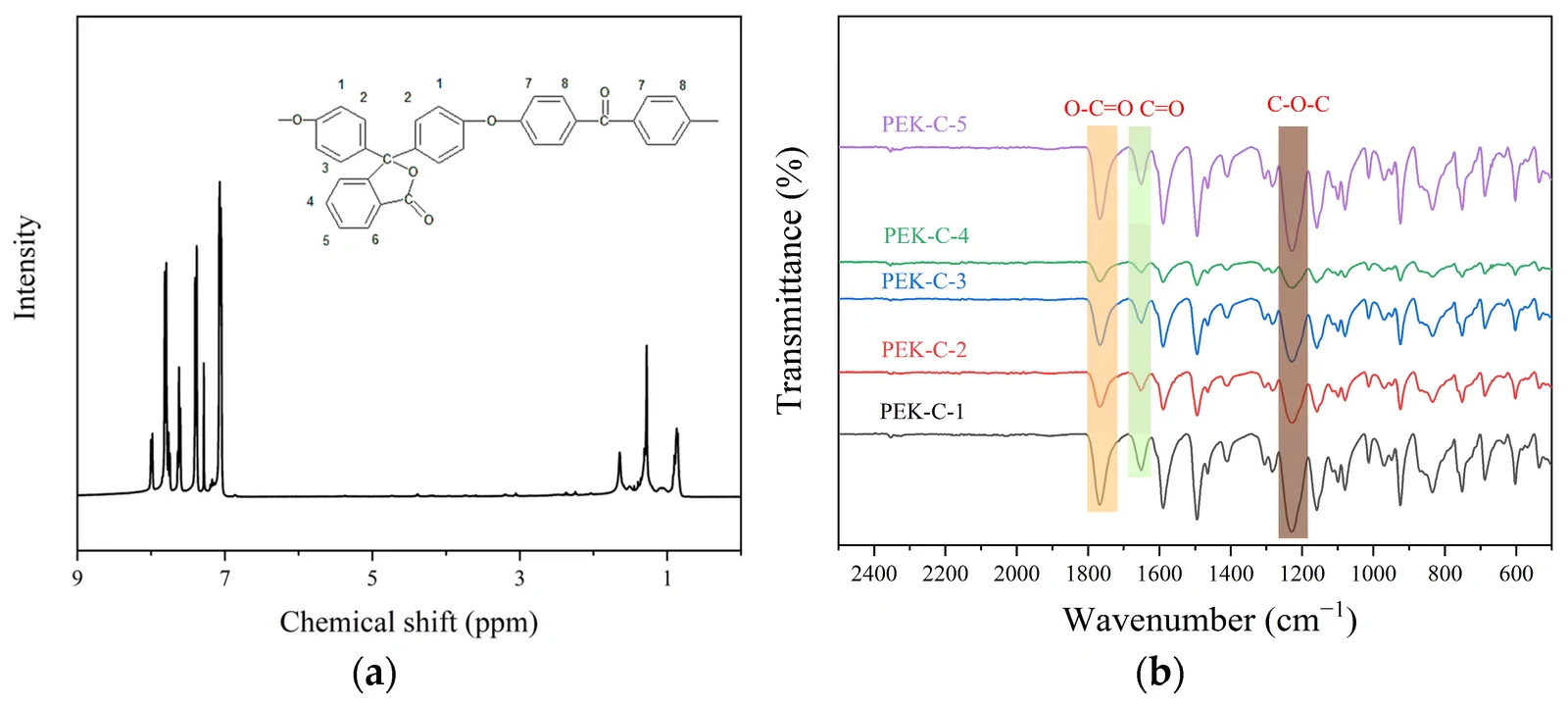
PEK-C polymers: (**a**) ^1^H NMR spectrum; (**b**) FT-IR spectra. The numerals on the chemical structure in (**a**) label protons with equivalent chemical environments.

The molecular structure of PEK-C was characterized by proton nuclear magnetic resonance (^1^H NMR) spectroscopy, and the results are shown in Figure 3a. The high symmetry of the PEK-C molecular structure and the overlap of aromatic proton peaks complicate the assignment of the relatively simple spectral lines. On this basis, the assignment of each proton signal peak was performed mainly based on the spectral line intensity in this study. The sum of the integrated proton numbers of all characteristic peaks in the full spectral range was calculated to be 20, which is completely consistent with the total number of hydrogen atoms in a single repeating unit of PEK-C. The ^1^H NMR results confirm the successful synthesis of the PEK-C polymer.

The molecular structure of the polymer was further characterized by Fourier-transform infrared (FT-IR) spectroscopy. The FT-IR spectra of PEK-C samples with different molecular weights are shown in Figure 3b. The analysis of the FT-IR spectrum showed that the absorption peak at 1230 cm^−1^ is attributed to the stretching vibration of Ar-O-Ar; the bands at 1495 cm^−1^ and 1590 cm^−1^ correspond to the skeleton vibration of the benzene ring; the peak at 1651 cm^−1^ is ascribed to the stretching vibration of C=O in the DFK group; and the peak at 1768 cm^−1^ originates from the stretching vibration of C=O in the PHT group. The FTIR results further verify the successful synthesis of the PEK-C polymer.

### 3.2. The Effect of PEK-C Molecular Weight on Flowability

The molecular weight and its distribution of PEK-C were characterized by gel permeation chromatography (GPC), and the results are summarized in Table 2. All PEK-C samples have a polydispersity index (PDI) of 1.46–1.51, indicating a relatively narrow molecular weight distribution. As the molar ratio of DFK to PHT increased from 1.044 to 1.056, the number-average molecular weight (Mn) of PEK-C decreased from 2.21 × 10^4^ to 2.01 × 10^4^, and the weight-average molecular weight (Mw) decreased from 3.32 × 10^4^ to 3.04 × 10^4^. GPC results demonstrate that the molecular weight of PEK-C can be regulated by adjusting the molar ratio of DFK to PHT during polymerization. Because an extreme reduction in the molecular weight would result in loss of PEK-C properties, the optimal molecular weight should be chosen according to the specific process conditions that are in place in the actual melt spinning process.

**Figure 4:**
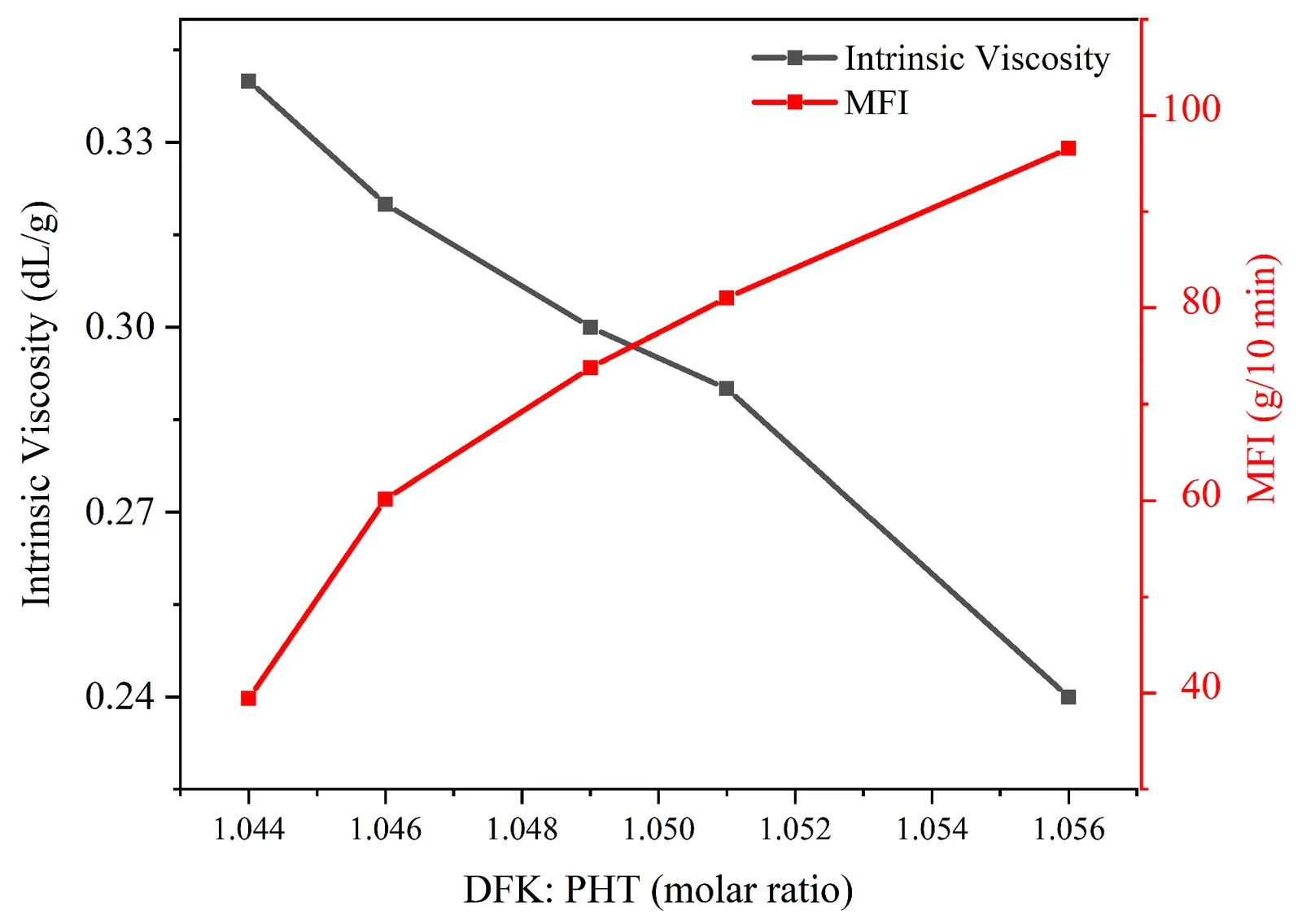
Flow properties of PEK-C at different molar ratios.

The flowability of the polymers was characterized by intrinsic viscosity and the melt flow index (MFI), and the results are listed in Figure 4. As the molar ratio of DFK to PHT increased from 1.044 to 1.056, the intrinsic viscosity of PEK-C decreased from 0.34 dL/g for PEK-C-1 to 0.24 dL/g for PEK-C-5. The MFI of PEK-C increased significantly with the increase in the molar ratio, from 39.45 g/10 min to 96.61 g/10 min. The presence of rigid aromatic rings and bulky phenolphthalein side groups in the PEK-C molecular chain leads to PEK-C exhibiting excellent heat resistance and mechanical properties but limits its melt flowability. Therefore, the precise control of PEK-C flowability by adjusting the molar ratio of DFK to PHT during polymerization is of great significance for melt processing. The results showed the flowability of PEK-C can be precisely regulated in a wide range by adjusting the molar ratio.

The growth of polymer molecular chains relies on the equimolar reaction between the monomers DFK and PHT. When the DFK monomer is in excess, PHT is consumed completely in advance, and the excess DFK acts as an end-capping agent in the late stage of the polymerization reaction, making the molecular chains lose reactivity, thus terminating the chain growth process. With the increase in the excess degree of DFK, the end-capping effect is enhanced, the relative molecular weight of the polymer decreases, and the entanglement degree of the molecular chains is significantly reduced. According to the Mark–Houwink equation [40],

(1) $$\left[\eta \right]=KM^{a}$$

In the equation, $\left[\eta \right]$ is the intrinsic viscosity; M is the molecular weight; within a certain range of molecular weights, K and a are constants. As the molecular weight decreased, the intrinsic viscosity of PEK-C decreased. Meanwhile, the lower molecular weight and reduced molecular chain entanglement degree significantly improve the mobility of the polymer molecular chains during melt processing, resulting in a substantial increase in the flowability of the PEK-C melt, which is finally manifested as a significant increase in MFI and a distinct improvement in flowability.

The molar ratio of monomers provides an important experimental basis for the melt spinning processing of PEK-C. Specifically, PEK-C resin with high flowability can be prepared by increasing the molar ratio of DFK to PHT, to meet the processing requirements for melt flowability.

### 3.3. Thermal Properties and Thermal Stability of PEK-C

During melt processing, too low a melting temperature results in inadequate flowability, which is not desirable in the processing and is unfavorable for processing, whereas too high a melting temperature can also cause thermal degradation of PEK-C, which reduces the mechanical properties. Therefore, it is important to choose the right range of melt processing temperatures based on the thermal properties and flowability of PEK-C.

**Figure 5:**
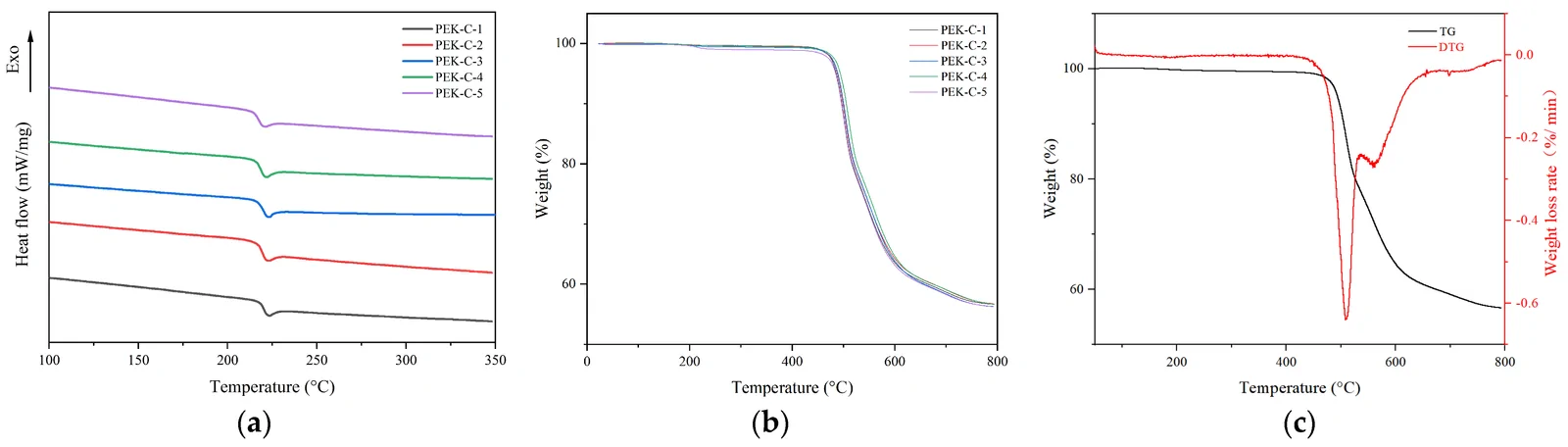
Thermal properties of PEK-C samples with different molecular weights: (**a**) DSC second heating curve; (**b**) TG curve; (**c**) TG-DTG curve.

The differential scanning calorimeter (DSC) and thermogravimetric analysis (TGA) were used to study the thermal properties of PEK-C, and the results are shown in Figure 5. Figure 5a presents the second heating DSC curves of PEK-C with different molecular weights. Figure 5a shows that all samples had an endothermic step of glass transition (Tg) at about 220 °C, and no obvious melting endothermic peak was detected, indicating that PEK-C is a typical amorphous polymer without a crystalline melting temperature (Tm). Figure 5b shows the Tg curves of PEK-C at different molecular weights. The results show that all PEK-C samples have a small weight loss rate at a temperature range from room temperature to 440 °C, which confirms the excellent thermal stability of PEK-C. When temperature exceeds 440 °C, all curves show a rapid weight loss step, which corresponds to the thermal degradation of the PEK-C molecular chain. At 800 °C, the weight loss rates of all PEK-C samples are 43.0–44.0%, exhibiting good thermal resistance. Figure 5c shows the TG-DTG curve of PEK-C-4. According to the TG curve, the weight loss rate of PEK-C is less than 1.0% in a temperature range of 50 °C to 440 °C, indicating the good thermal stability of PEK-C. When the temperature exceeded 440 °C, the mass loss rate decreased significantly, and the weight loss rate reached 56.6% when the temperature was further raised to 800 °C, showing good heat resistance. The DTG curve indicates that the thermal decomposition rate of PEK-C increases significantly as the temperature rises to 440 °C, and the highest rate of thermal decomposition is reached at 520 °C, indicating that the molecular chain of PEK-C is significantly degraded at 520 °C.

The glass transition temperature (Tg) and 5% weight loss rate (Td_5%_) of PEK-C with different molecular weights are listed in Table 3. The results show that the glass transition temperatures of all PEK-C samples are near 220 °C; the high Tg of PEK-C arises from the aromatic rings, carbonyl groups and PHT groups of molecules, which hinder the segmental motion of molecular chains. Moreover, the Tg of PEK-C only decreased slightly with the reduction in the polymer molecular weight. The reason is that the mobility of polymer molecular chains is mainly affected by the end groups, which account for an extremely low proportion in long polymer chains. As an amorphous polymer, the minimum temperature of PEK-C for melt processing is set to Tg + 100 °C, which is 320 °C.

During melt processing, to avoid thermal decomposition of the polymer due to long-term residence in the processing equipment and the resulting performance degradation, the processing temperature must be much lower than Td_5%_ to reserve a sufficient safety margin. As shown in Table 3, with the decrease in PEK-C molecular weight, the Td_5%_ only shows a slight fluctuation, ranging from 484.0 °C to 493.9 °C. The reason is that most thermal decomposition reactions of polymers are initiated from the thermally unstable chain end sites, which account for an extremely low proportion in long polymer chains, and the proportion of thermal degradation initiated by end groups is negligible. Therefore, the molecular weight of PEK-C has a negligible effect on Td_5%_.

During melt processing, it was found that PEK-C undergoes a thermal crosslinking degradation reaction at a temperature much lower than Td_5%_. The molecular chains are interconnected to form a crosslinked network structure, which transforms PEK-C from thermoplastic to thermosetting. Thermal decomposition crosslinking reactions of PEK-C have been researched in prior studies [41,42]. During the thermal decomposition crosslinking of PEK-C, the intracycle ester bonds (O–C=O) on the phenolphthalein side chains undergo cleavage at high temperatures, generating oxygen radicals and carboxyl groups. The unstable carboxyl groups are eliminated first and subsequently react with partially released oxygen radicals to form carbon monoxide (CO) and carbon dioxide (CO_2_). The carbon atoms exposed upon the release of oxygen radicals combine with the unreacted oxygen radicals on adjacent molecular chains, forming carbon–oxygen bridges that induce crosslinking between the polymer chains.

By regulating the degree of the thermal crosslinking degradation reaction, PEK-C nanofiltration membranes with different gas separation properties can be fabricated. But it is unfavorable for melt spinning, because the thermal crosslinking degradation reaction will lead to a significant decrease in melt flowability.

**Figure 6:**
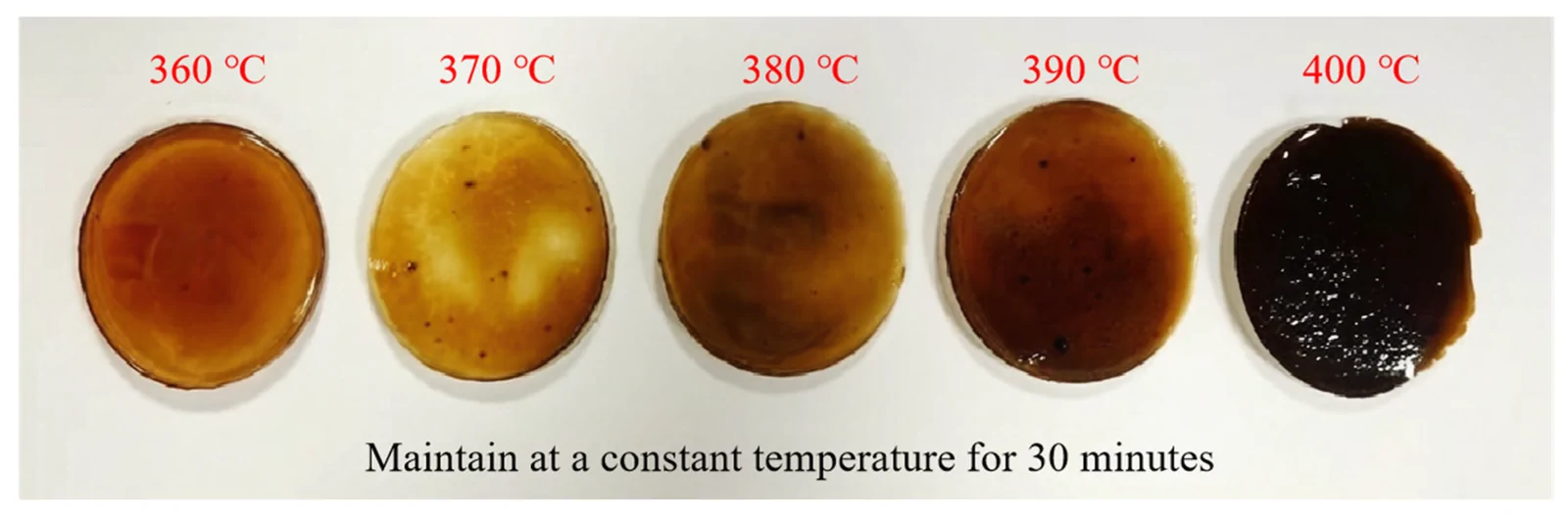
Changes in PEK-C after heating for 30 min at different temperatures.

PEK-C samples (45 mm in length and 2 mm in thickness) were placed in a muffle furnace and held at 360, 370, 380, 390, and 400 °C for 30 min in an air atmosphere. The changes in PEK-C after heating for 30 min at different temperatures are shown in Figure 6. When the temperature is lower than 370 °C, PEK-C maintains a uniform and transparent color. When the temperature is higher than 380 °C, the degree of carbonization and blackening of PEK-C increase with the rise in temperature. In addition, the gas released by thermal decomposition is trapped inside the melt to form bubbles due to the viscosity and surface tension of the melt, and the number of bubbles increases with the increase in temperature.

**Figure 7:**
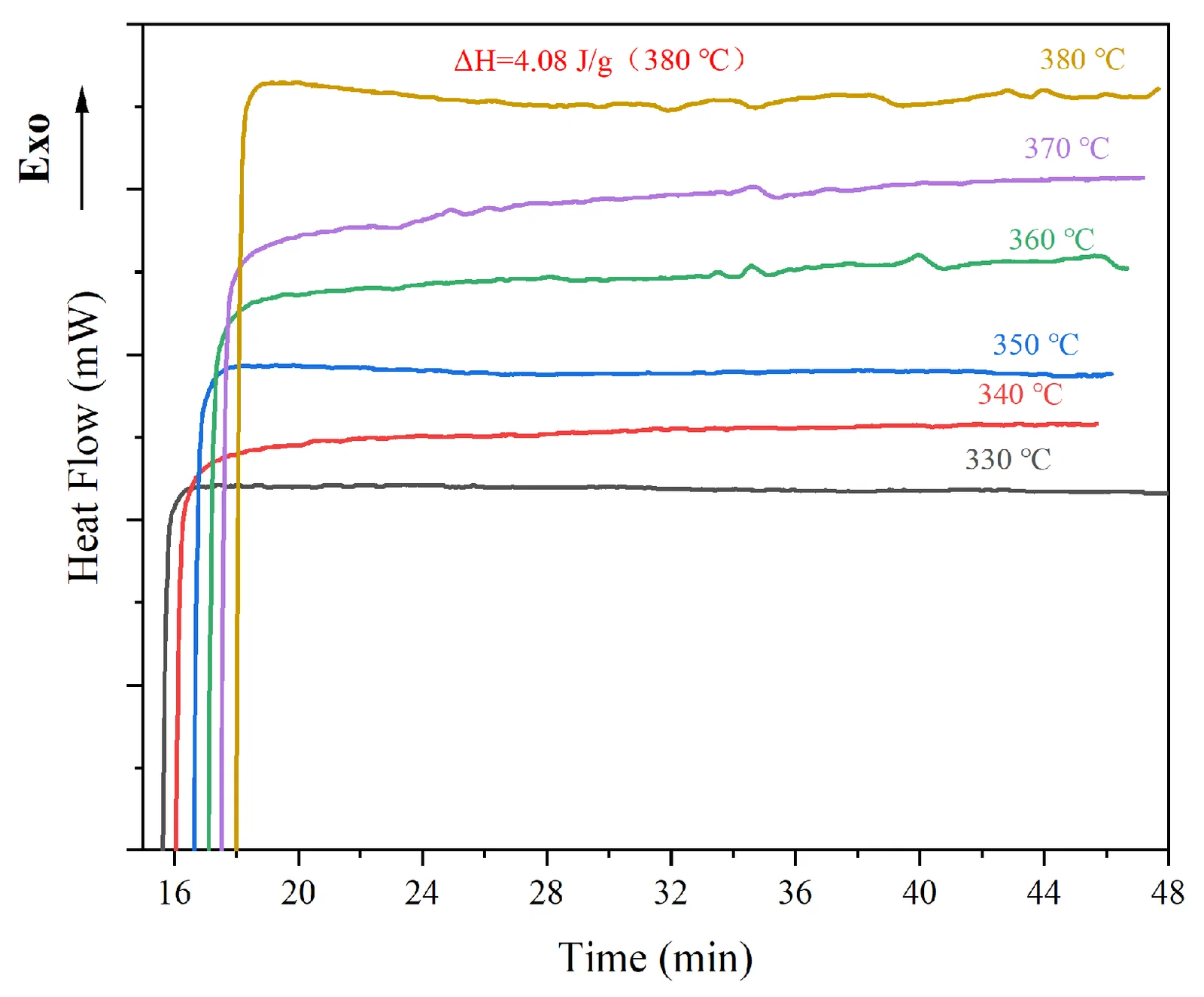
DSC curves for PEK-C after being held at constant temperature for 30 min at different temperatures.

Figure 7 shows the DSC curves of PEK-C during isothermal keeping for 30 min in a nitrogen atmosphere after heating to different temperatures. The results show that when the isothermal temperature is 330 °C, 340 °C, 350 °C, 360 °C, and 370 °C, the DSC curve quickly stabilizes during the isothermal process after the heat flow’s abrupt change from the heating stage to the isothermal stage, and no obvious characteristic endothermic or exothermic peaks appear during the entire 30 min isothermal period. The results indicate that, in the temperature range of 330 °C to 370 °C, no significant chemical reactions such as thermal decomposition, crosslinking, and depolymerization occur. PEK-C maintains excellent thermal stability under long-term isothermal conditions, with no obvious deterioration of properties, which can meet the requirements of melt spinning processing for melt thermal stability. When the isothermal temperature rises to 380 °C, the DSC curve shows a significant shift to the endothermic direction during the isothermal stage, presenting a continuous endothermic effect. The total enthalpy change ΔH during the isothermal process is calculated to be 4.08 J/g by integration. As an amorphous thermoplastic resin, PEK-C has no crystalline melting-phase transition process. Therefore, this continuous endothermic signal does not originate from physical phase transition but corresponds to the endothermic reaction of thermal crosslinking degradation of the polymer. Therefore, the temperature during melt processing of PEK-C must not exceed 380 °C.

During melt processing, PEK-C was fed into the processing equipment and heated rapidly to a constant temperature. The residence time in the equipment is less than 20 min, which is highly consistent with the test procedure of rapid heating to a constant temperature followed by isothermal holding for 30 min in the DSC test. Therefore, the isothermal DSC test results are more consistent with the actual thermal history and can more accurately reflect the thermal stability of the material in the actual processing scenario.

The results of the above experiment prove that PEK-C has no significant thermal degradation within 30 min at 370 °C, and its thermal stability fully meets melt processing requirements. The lowest temperature for melt processing of PEK-C is determined to be 320 °C based on the previous DSC test results. The optimal melt processing temperature range of PEK-C is 320–370 °C. At such a temperature range, not only can PEK-C melts possess adequate flow ability and formability, but they will also not experience any thermal degradation and thermal crosslinking degradation reactions during melt processing.

### 3.4. Rheological Properties of PEK-C

During melt spinning, the polymer melt undergoes directional flow under the action of shear force, and its rheological behavior directly determines the continuity of melt spinning, the uniformity of fiber diameter, and the final molding properties of the fiber, which is the core basis for evaluating the spinnability of materials. The melt rheological behavior of polymer melt at high shear rates in capillary rheological tests is highly consistent with that in the melt spinning process. Therefore, in this study, a capillary rheometer was used to investigate the effects of molecular weight and melt processing temperature on the shear rheological behavior of PEK-C melts. Combined with key rheological parameters including non-Newtonian index, viscous flow activation energy and structural viscosity, this work provides theoretical and experimental support for the melt spinning of PEK-C.

**Figure 8:**
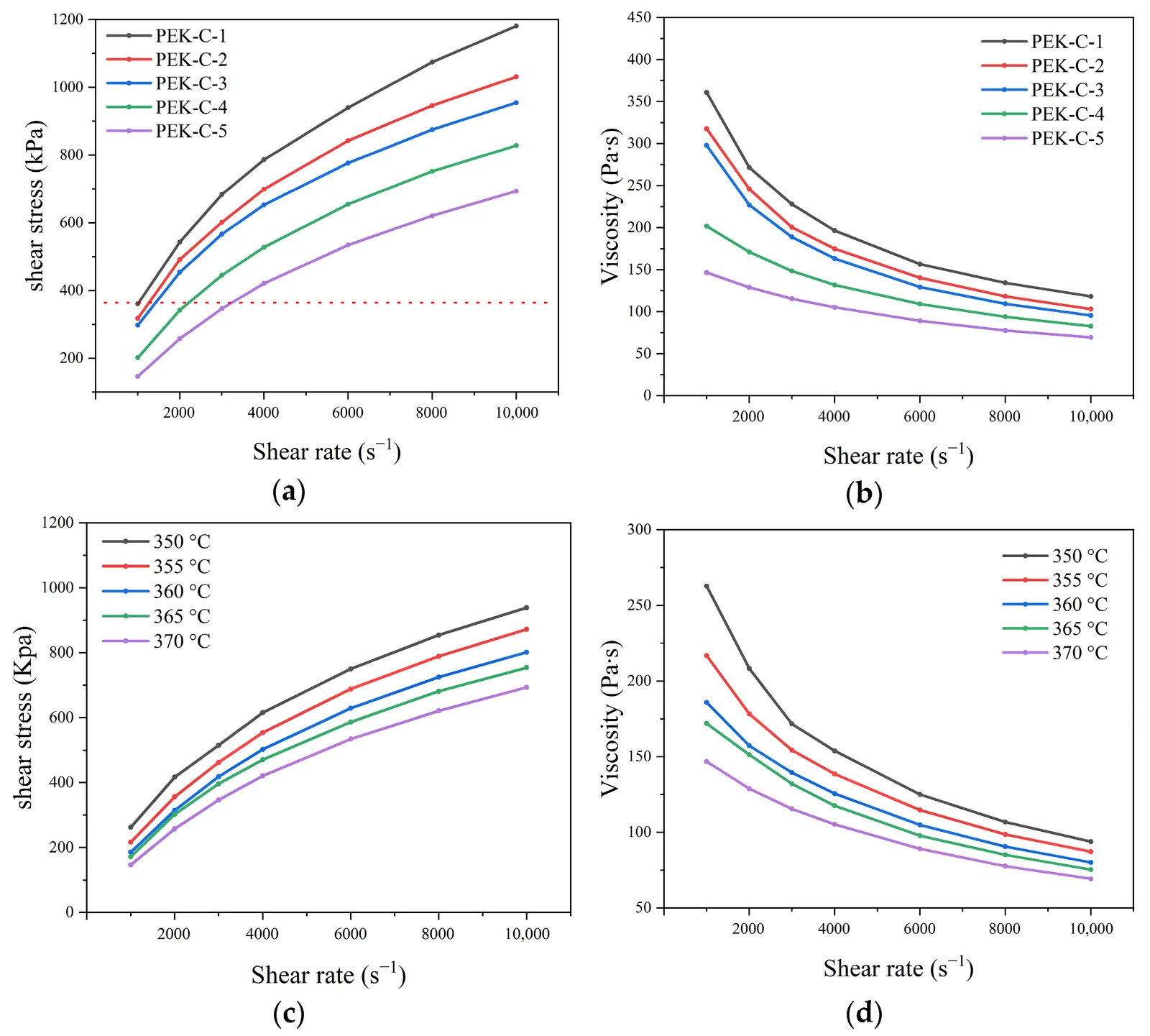
Capillary rheological properties of PEK-C: (**a**) the curves of shear stress versus shear rate for PEK-C with different molecular weights; (**b**) the curves of apparent shear viscosity versus shear rate for PEK-C with different molecular weights; (**c**) the curves of shear stress versus shear rate for PEK-C-5 at different temperatures; (**d**) the curves of shear viscosity versus shear rate for PEK-C-5 at different temperatures. The red dashed line indicates the maximum allowable melt shear stress of 375 kPa corresponding to the maximum safety pressure of the equipment. PEK-C samples with shear stresses below 375 kPa meet the safety requirements for extrusion.

Figure 8a shows the curves of shear stress versus shear rate for PEK-C with different molecular weights. In the shear rate range of 1000–10,000 s^–1^, the shear stress of all PEK-C samples exhibited a monotonically increasing trend with the increase in shear rate, showing significant non-Newtonian flow characteristics. At the same shear rate, the shear stress decreased with the reduction in PEK-C molecular weight. This is attributed to the increase in polymer molecular weight, leading to longer molecular chain length and a higher entanglement degree of molecular chains, which raises the flow resistance that needs to be overcome during the melt flow, thus manifesting as higher shear stress.

To achieve continuous and stable operation of melt spinning, the melt is required to have sufficient fluidity in the shear rate range of 3000–6000 s^−1^, and the extrusion pressure shall not exceed the safety upper limit of 15 MPa. According to the Stokes equation [43],

(2) $$\Delta p\cdot \pi R^{2}=2\pi R\cdot L\cdot \tau_{w}$$

where Δp is the extrusion pressure (Pa), R is the radius of the capillary die (mm), L is the length of the capillary die (mm), and $\tau_{w}$ is the shear stress at the capillary wall (Pa).

The diameter–length ratio of the capillary die is L/R = 5/0.25, its length is L = 5 mm and its radius is R = 0.25 mm. The calculation shows that the largest permissible shear stress of the melt corresponding to the maximum safety pressure of the equipment is 375 kPa. In the shear rate range of 3000–6000 s^–1^, only sample PEK-C-5 has a shear stress of 346 kPa at 3000 s^–1^, which meets the safety extrusion pressure requirement. The shear stress of other PEK-C samples exceeds the upper safety limit of the equipment, making it impossible to achieve melt spinning. Figure 8b presents the curves of apparent shear viscosity versus shear rate for PEK-C with different molecular weights. In the shear range of 1000–10,000 s^−1^, the apparent shear viscosity of all PEK-C samples decreased with the increase in shear rate, showing typical shear-thinning behavior. This is because at low shear rates, PEK-C molecular chains are in a thermodynamic equilibrium state of random entanglement, resulting in high viscosity. With the increase in shear rate, the external force from the shear flow field causes the oriented movement of molecular chains along the flow direction and the disentanglement of molecular chains, which ultimately manifests as a continuous decrease in apparent shear viscosity with the increase in shear rate. At the same shear rate, the apparent shear viscosity decreased with the reduction in molecular weight, which is consistent with the variation law of shear stress.

Melt processing temperature is the key parameter regulating the fluidity of polymer melts. Limited by the thermal crosslinking degradation reaction of PEK-C, five temperature points (350, 355, 360, 365 and 370 °C) were selected in this study to conduct capillary rheological tests on PEK-C-5, and the results are shown in Figure 8c,d.

Figure 8c shows the curves of shear stress versus shear rate for PEK-C-5 at different melt temperatures. In the temperature range of 350–370 °C, the shear stress of PEK-C-5 increased with the increase in shear rate. At the same shear rate, the shear stress of PEK-C-5 melt decreased significantly with the increase in melt temperature. This is because the rise in temperature enhances the mobility of molecular chain segments and reduces the entanglement degree of molecular chains. Figure 8d displays the curves of shear viscosity versus shear rate for PEK-C-5 at different melt temperatures. The shear viscosity of PEK-C-5 decreased with the increase in shear rate, showing shear-thinning behavior. At the same shear rate, the apparent shear viscosity of the melt decreased with the rise in melt temperature, which is consistent with the variation law of shear stress.

According to the capillary rheological test results, reducing the molecular weight of the polymer and increasing the melt processing temperature can decrease the shear viscosity of PEK-C melt, thereby meeting the process requirements of melt spinning. The melt processing temperature is limited by the thermal crosslinking degradation reaction and shall not exceed 380 °C, so the optimal melt processing temperature is determined to be 370 °C. Although reducing the molecular weight of the polymer can lower the shear viscosity of the melt, an excessive reduction in molecular weight will lead to the deterioration of the mechanical properties of the polymer. PEK-C-5, with the smallest molecular weight in all the samples, just meets the extrusion pressure and fluidity requirements of melt spinning at a melt processing temperature of 370 °C and shear rate of 3000 s^−1^.

The non-Newtonian index (n) can characterize the degree of deviation of PEK-C melt from Newtonian fluid behavior. The closer n is to 1, the closer the melt flow characteristics are to Newtonian fluid, which means it is easier to achieve stable control of the spinning process. For pseudoplastic non-Newtonian fluids, the relationship between shear stress and shear rate follows the Ostwald equation:

(3) $$\tau_{w}=K\gamma^{n}$$

where $\tau_{w}$ is the shear stress at the capillary wall of the melt (Pa), n is the non-Newtonian index, K is the consistency coefficient representing the consistency of the non-Newtonian fluid, and γ is the shear rate (s^−1^).

**Figure 9:**
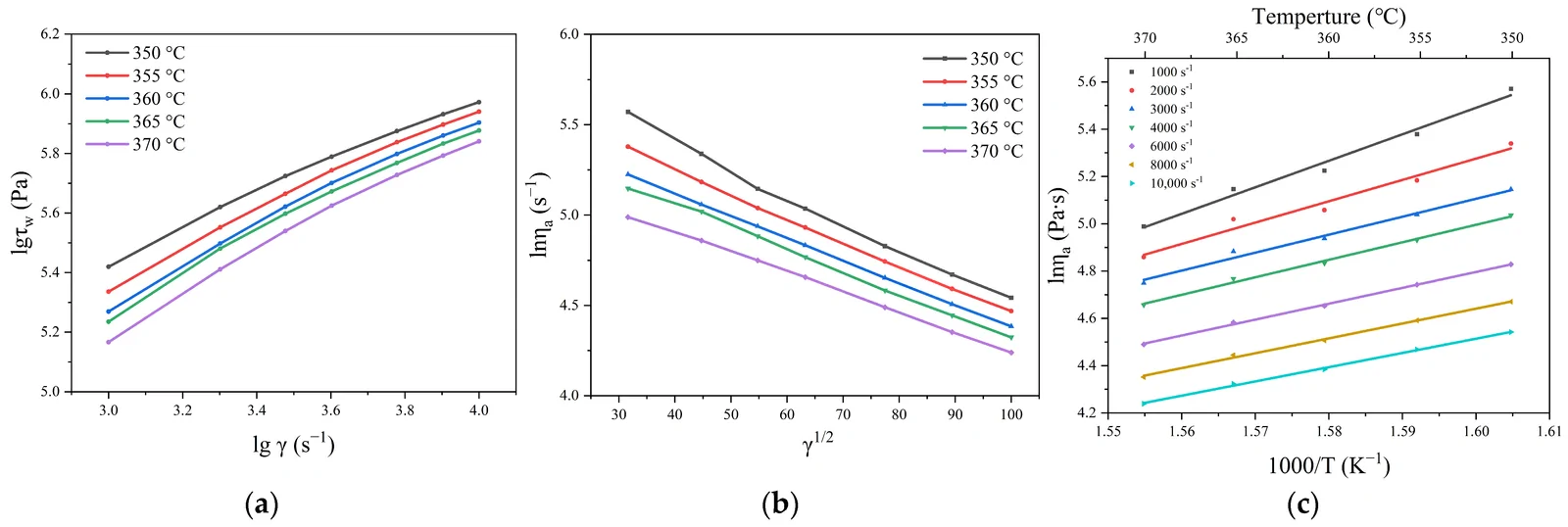
Rheological parameters of PEK-C-5: (**a**) the curves of lg$\tau_{w}$ versus lgγ for PEK-C-5 at different melt temperatures; (**b**) the curves of ln$\eta_{a}$ versus $\gamma^{1/2}$ for PEK-C-5 melt at different melt temperatures; (**c**) the curves of ln$\eta_{a}$ versus 1000/T for PEK-C-5 at different shear rates.

Figure 9a shows the curves of lg$\tau_{w}$ versus lgγ for PEK-C-5 at different melt temperatures, and the slope of the fitted straight line is the non-Newtonian index. The results of PEK-C-5 at different melt temperatures are listed in Table 4. Within the temperature range of 350–370 °C, the non-Newtonian indexes of PEK-C melt ranges from 0.55 to 0.67, all are lower than 1, indicating that the PEK-C melt exhibits typical pseudoplastic fluid behavior. This arises because shear force tends to break molecular chain entanglements at high shear rates, enhancing flowability. The value of n increases with rising temperature, and this phenomenon is attributed to the enhanced mobility of polymer chain segments at elevated temperatures: improved chain mobility weakens intermolecular chain entanglement and reduces melt viscosity, which in turn facilitates shear flow of the melt. This is conducive to reducing the problems such as uneven fiber diameter and filament breakage caused by shear rate fluctuations during spinning and improving the stability of the spinning process. Therefore, in the spinning process, the flow properties of the melt can be controlled by adjusting the shear rate and melting temperature.

The structural viscosity index (Δη) is an important parameter characterizing the degree of entanglement and structuring degree of molecular chains in the melt. For pseudoplastic fluids with shear-thinning behavior, Δη > 0. A smaller Δη indicates a lower entanglement degree of molecular chains and a weaker structuring degree of the melt, thus leading to better spinnability and stability of the melt. The calculation formula of the structural viscosity index Δη is as follows:

(4) $$\Delta \eta =-\left(\frac{d\gamma^{1/2}}{d{\ln\eta }_{a}}\right)\times 10^{2}$$

Figure 9b shows the curves of ln$\eta_{a}$ versus $\gamma^{1/2}$ for PEK-C-5 melt at different melt temperatures. After calculations, the negative value of the slope of the straight line is Δη. The calculated results of the structural viscosity index of PEK-C-5 at different temperatures are listed in Table 4. According to the results, the Δη of PEK-C-5 melt shows a continuous downward trend with the rise in temperature, indicating that increasing the temperature can effectively reduce the entanglement degree between molecular chains and significantly improve the spinnability of the material. The high entanglement degree of molecular chains caused by the rigid aromatic rings in the main chain and the bulky phenolphthalein side groups of PEK-C is the core reason for the poor spinnability of PEK-C. Increasing the melt temperature to 370 °C can effectively reduce the entanglement degree of molecular chains, providing favorable conditions for continuous and stable spinning.

At the same shear rate, the viscous flow activation energy ($E_{\eta }$) is a key parameter characterizing the sensitivity of polymer melt viscosity to temperature changes. The larger the $E_{\eta }$, the more significant the influence of temperature fluctuations on melt viscosity, and the higher the precision requirement for temperature control during spinning. When the melt processing temperature is higher than the viscous flow temperature of the polymer (Tg + 100 °C), the relationship between the shear viscosity of the polymer and temperature follows the Arrhenius empirical formula:

(5) $$\eta_{a}=A\cdot \exp\left(\frac{E_{\eta }}{R\cdot T}\right)$$

where $\eta_{a}$ is the apparent shear viscosity (Pa·s), A is the pre-exponential factor, $E_{\eta }$ is the viscous flow activation energy (J/mol), R is the gas constant (8.314 J/(mol·K)), and T is the absolute temperature (K).

Figure 9c shows the curves of ln$\eta_{a}$ versus 1000/T for PEK-C-5 at different shear rates. The $E_{\eta }$ of PEK-C-5 at different shear rates is calculated by fitting the slope of the straight line, and the results are listed in Table 5. The results show that PEK-C-5 exhibits a high viscous flow activation energy ($E_{\eta }$) of 93.20 kJ/mol at a low shear rate of 1000 s^−1^. This indicates that its viscosity is relatively sensitive to temperature changes. This is attributed to the presence of rigid aromatic rings in the main chain of PEK-C, as well as the bulky phenolphthalein side groups, which reduce the flexibility of the molecular chains and result in an obvious steric hindrance effect. However, as the shear rate increases, $E_{\eta }$ shows a pronounced downward trend. In the spinning interval with a shear rate of 3000–6000 s^−1^, $E_{\eta }$ stabilizes at 55–63 kJ/mol, indicating that under high shear action, the entanglement degree of polymer molecular chains decreases. Consequently, less energy is required for molecular segments to overcome the energy barrier, and the influence of temperature on melt viscosity is weakened. This improves the error tolerance to temperature fluctuations and is conducive to the stability of the spinning process.

Based on the above rheological result, the optimal process parameters for PEK-C melt spinning are finally determined. PEK-C-5 is selected for melt spinning because its melt has low viscosity, flow characteristics closest to Newtonian fluid and the lowest structural viscosity index. The optimal spinning temperature is 370 °C. At this temperature, PEK-C has good thermal stability and low viscosity.

### 3.5. Study on the Spinnability of PEK-C and Investigation of Spinning Processes

**Figure 10:**
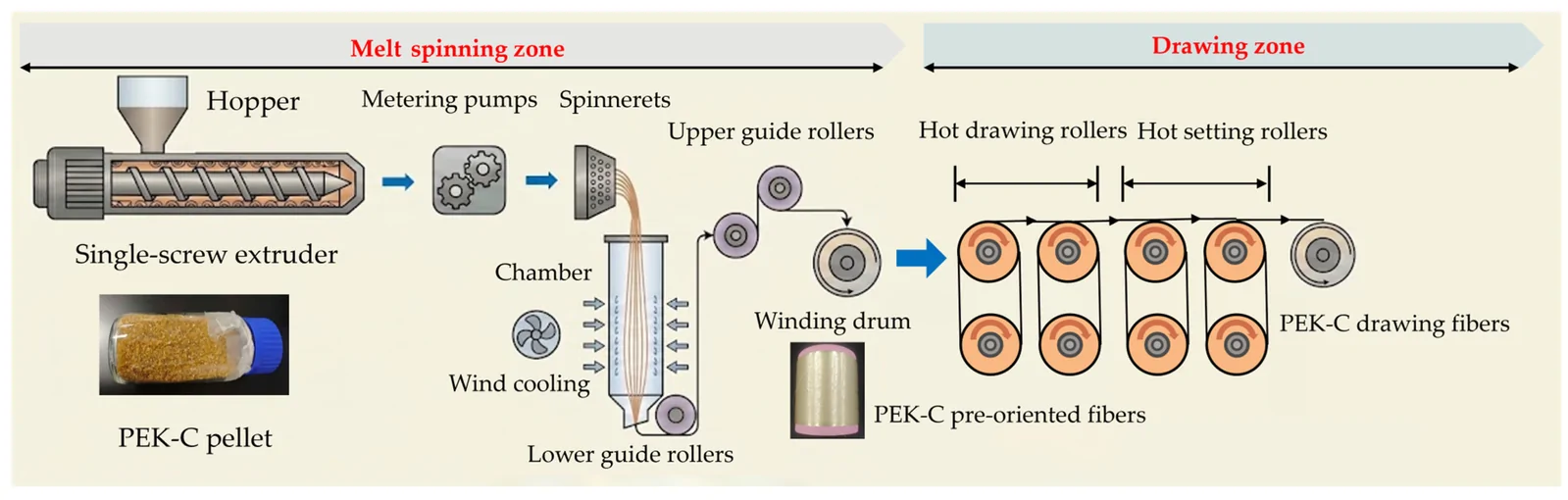
Flowchart of PEK-C fiber-forming processes.

A flowchart of the PEK-C fiber-forming processes is shown in Figure 10. Prior to spinning, PEK-C pellets were fed into the extruder without installing the spinning pack, and the melt was extruded at a low screw speed for material testing and purging. PEK-C-1 and PEK-C-2 showed no spinnability, as their excessively high melt viscosity prevented the extrudate from forming continuous filaments. PEK-C-3 and PEK-C-4 could form filamentous extrudates, but the filaments were prone to breakage with non-uniform diameters, indicating poor spinnability. PEK-C-5 produced continuous fibers upon extrusion, confirming that its molecular weight and melt viscosity were optimal for spinning.

Fibers were subsequently prepared using PEK-C-5 with the spinning pack installed. Following the experimental procedure detailed in Section 2.3, uncollected as-spun fibers and pre-oriented fibers were obtained. The pre-oriented fiber samples are designated according to the rotational speed of the metering pump. PO-24 rpm and PO-21 rpm fibers correspond to the fibers spun at metering pump speeds of 24 rpm and 21 rpm, respectively.

The drawing process is shown in Figure 10. The hot drawing rollers affect fiber drawing via the linear speed differential between the preceding and subsequent roller pairs, with the linear speed of the second pair of hot drawing rollers being higher than that of the first pair. The linear speed of the hot setting rollers is slightly lower than that of the drawing rollers, permitting a minor shrinkage of 1–5% in the fibers. This process eliminates the residual internal stress induced by hot drawing and markedly enhances the dimensional stability and thermal resistance of the fibers.

**Figure 11:**
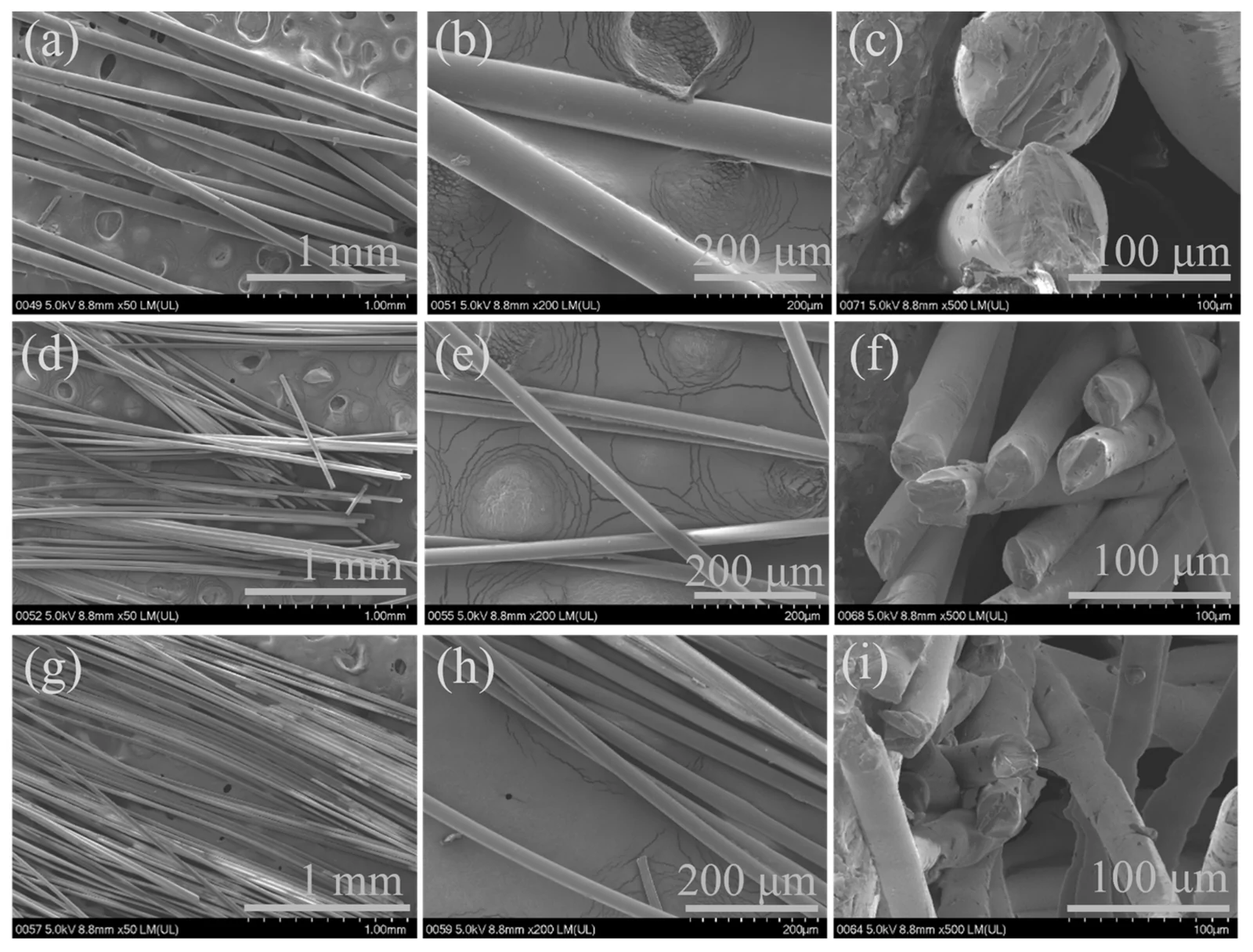
SEM images of PEK-C fibers: (**a**) as-spun fibers; (**b**) close-up of as-spun fibers; (**c**) cross-section of as-spun fibers; (**d**) pre-oriented fibers with draw speed; (**e**) close-up of pre-oriented fibers; (**f**) cross-section of pre- oriented fibers; (**g**) pre-oriented fibers; (**h**) close-up of pre-oriented fibers; (**i**) cross-section of pre- oriented fibers.

Figure 11 shows the surface and cross-sectional morphologies of PEK-C fibers. At low magnification, all three samples are continuous fibers with favorable overall dispersion, and no severe agglomeration or adhesion is observed. This indicates that no obvious filament merging or melt adhesion occurred during the post-spinning treatment. At high magnification, the surfaces of all three types of fibers are generally smooth without distinct grooves, cracks or other defects. This result demonstrates that the melt spinning process of PEK-C is stable with good melt homogeneity, and no notable spinneret fouling, melt fracture or surface flaws arise during met spinning. As revealed by cross-sectional morphologies of PEK-C fibers, the internal structures of all three fibers are dense, and no visible pores are observed. The result shows that the fibers achieve full densification during the melt spinning process, with no defects such as entrapped bubbles or inhomogeneous melting.

Based on the acquired SEM images, quantitative statistical analysis of fiber diameter and its distribution was performed using ImageJ image processing software, with no fewer than 20 filaments counted for each sample. The results are summarized in Table 6. The average diameters of the PEK-C as-spun, PO-21 rpm, and PO-24 rpm fibers are 90.71 μm, 27.23 μm and 30.43 μm, respectively. The average diameter of the PEK-C as-spun fibers is significantly larger than that of the PO-21 rpm and PO-24 rpm processed via a high-speed wind drum. This is because the PEK-C as-spun fibers are undrawn fibers formed by the natural cooling and solidification of melt. In contrast, for the PO-21 rpm and PO-24 rpm fibers produced at a wind drum speed of 600 m/min, PEK-C fibers are subjected to sustained axial tensile stress during cooling and solidification, which induces irreversible plastic deformation and molecular chain orientation. As a result, the axial dimension of the fibers increases substantially, accompanied by a marked refinement of the radial diameter.

The PEK-C as-spun fibers exhibit a diameter standard deviation of 8.00 μm, a coefficient of variation (CV) of 8.82%, and a diameter distribution ranging from 86 to 94 μm, indicating a low degree of diameter dispersion and favorable diameter uniformity. This is because of the absence of stress fluctuations caused by high-speed drawing during the formation of as-spun fibers; the melt solidifies steadily under natural cooling, leading to minor diameter variations. In comparison, the PO-21 rpm and PO-24 rpm fibers processed by the high-speed wind drum show diameter standard deviations of 3.76 μm and 4.95 μm, coefficients of variation of 13.80% and 16.27%, and diameter distribution ranges of 21–35 μm and 22–40 μm, respectively. Their relative degree of diameter dispersion is higher than that of the as-spun fibers. This arises because the extensional rheological behavior of the melt, fluctuations in temperature, air velocity in the air-cooling field, and tension fluctuations in the winding system all impose disturbances on the solidification and shaping process of the fibers, thereby causing a slight increase in diameter dispersion. Nevertheless, the diameter CV values of PO-21 rpm and PO-24 rpm samples are below 20%, with no extreme abnormal diameter values observed. This demonstrates that the melt spinning process established in this study maintains favorable stability, and the diameter uniformity of the resultant fibers can meet the experimental requirements for subsequent structural and property characterization.

This study investigates the mechanical properties and molecular chain orientation of PEK-C at different drawing ratios, revealing the regulation mechanisms of fiber structure and properties during the spinning–drawing process. PEK-C pre-oriented fibers were subjected to drawing at different draw ratios, yielding three sets of PEK-C fibers with draw ratios of 1.5, 2.0 and 2.5, designated as DR-1.5, DR-2.0 and DR-2.5.

Their mechanical properties were characterized via tensile testing, and the tensile properties of PEK-C fibers are summarized in Table 7. All numerical values are reported as the means of no fewer than five independent replicate measurements. Specifically, the as-spun PEK-C fibers exhibited a tensile strength of merely 0.367 cN/dtex and a breaking strength of 0.490 cN/dtex. With an increasing draw ratio, the tensile strength of the drawn PEK-C fibers increased from 0.499 cN/dtex to 0.560 cN/dtex, while the breaking strength rose from 0.643 cN/dtex to 1.059 cN/dtex. Both properties showed a continuous upward trend with increasing draw ratio. This demonstrates that hot drawing treatment exerts a significant optimization effect on the mechanical properties of PEK-C fibers.

PEK-C exhibits poor melt spinnability due to its high degree of molecular chain entanglement. In this work, continuous melt spinning of PEK-C was successfully achieved by reducing the molecular weight and optimizing the melt spinning temperature. However, the substantial reduction in molecular weight leads to relatively inferior mechanical properties of the resultant PEK-C fibers, which are comparable to those of solution-spun PEK-C fibers documented in the existing literature [32]. Accordingly, further optimization of the melt spinning process parameters is required in subsequent studies to enhance the mechanical performance of PEK-C fibers.

Birefringence measurements were performed on PEK-C fibers with different draw ratios, and the results are presented in Table 8. The as-spun PEK-C fibers showed no effectively detectable birefringence signal because as-spun fibers are undrawn fibers formed by natural cooling and solidification of melt filaments, in which the internal molecular chains are in a randomly coiled and entangled state with no significant preferential orientation along the fiber axis. The birefringence Δn of PEK-C fibers increased from 0.07 to 0.09 with an increasing draw ratio. This structural feature is consistent with the low mechanical strength of as-spun fibers: randomly oriented molecular chains cannot achieve efficient axial stress transfer, resulting in poor load-bearing capacity of the fibers. The increase in Δn of PEK-C fibers with draw ratio indicates that the tensile stress along the fiber axis can induce disentanglement and axial orientation of PEK-C molecular chains, and the degree of orientation continuously improves with increasing draw ratio.

Combined with the mechanical property results, the increasing trend of Δn of PEK-C fibers is completely consistent with the rising trends of tensile strength and breaking strength with draw ratio and corresponds to the decreasing trend of elongation at break. During drawing, the high axial orientation of molecular chains enables efficient stress transfer along the rigid molecular backbones under external force, fully exploiting the intrinsic mechanical properties of PEK-C polymer and ultimately leading to a significant improvement in fiber strength. Meanwhile, the conformational space available for movement of highly oriented molecular chains is greatly restricted, reducing the plastic deformation capacity of the fibers. Consequently, the elongation at break decreased as the degree of orientation increased. The birefringence test results of this study reveal the core mechanism by which hot drawing regulates the mechanical properties of PEK-C fibers at the aggregated state structure level.

## 4. Conclusions

In summary, continuous melt spinning of PEK-C fibers was successfully achieved through rheology modulation and melt strength enhancement. By adjusting the monomer ratio, a series of PEK-C resins with gradient molecular weights were synthesized, enabling precise control of melt viscosity while maintaining the thermal stability and mechanical properties. The PEK-C with a molecular weight of 2.01 × 10^4^ g mol^–1^ exhibited suitable melt fluidity and processability, and the optimal spinning temperature was determined to be 370 °C. Further compounding of as-polymerized PEK-C powder into pellets allows stable and continuous melt spinning for fiber fabrication. The obtained PEK-C fibers exhibited tensile strength and breaking strength of 0.56 and 1.06 cN dtex^–1^, respectively. Moreover, the relationship between spinning parameters, mechanical properties, and molecular chain orientation was further clarified, providing guidance for optimizing the melt spinning process of PEK-C fibers. This work establishes a feasible strategy for the continuous and green manufacturing of PEK-C fibers, which is expected to resolve the environmental contamination and low productivity issues associated with traditional solution spinning and electrospinning.

## Figures and Tables

**Table 1:** Control of the molar ratio in the PEK-C polymerization reaction.

Polymer	DFK Weight (g)	PHT Weight (g)	DFK:PHT (Molar Ratio)
PEK-C-1	30.00	41.92	1.044
PEK-C-2	30.00	41.82	1.046
PEK-C-3	30.00	41.72	1.049
PEK-C-4	30.00	41.64	1.051
PEK-C-5	30.00	41.44	1.056

**Table 2:** Molecular weight and distribution of PEK-C polymers.

Polymer	DFK:PHT (Molar Ratio)	Mn (g/mol)	Mw (g/mol)	PDI
PEK-C-1	1.044	2.21 × 10^4^	3.32 × 10^4^	1.50
PEK-C-2	1.046	2.15 × 10^4^	3.21 × 10^4^	1.49
PEK-C-3	1.049	2.10 × 10^4^	3.07 × 10^4^	1.46
PEK-C-4	1.051	2.05 × 10^4^	3.05 × 10^4^	1.48
PEK-C-5	1.056	2.01 × 10^4^	3.04 × 10^4^	1.51

**Table 3:** Tg and Td_5%_ of PEK-C.

Polymer	Tg (°C)	Td_5%_ (°C)
PEK-C-1	221.1	486.0
PEK-C-2	220.5	488.3
PEK-C-3	219.7	489.2
PEK-C-4	219.0	493.9
PEK-C-5	217.2	484.8

**Table 4:** The n and Δη coefficients of PEK-C-5 at different temperatures.

Temperature (°C)	n	Δη
350	0.55	2.01
355	0.59	1.90
360	0.62	1.83
365	0.63	1.78
370	0.67	1.75

**Table 5:** The $E_{\eta }$ coefficients of PEK-C-5 at different temperatures.

Shear Rate (s^–1^)	$E_{\eta }$ (kJ/mol)
1000	93.20
3000	75.01
3000	63.14
4000	61.54
6000	55.85
8000	52.26
10,000	50.10

**Table 6:** Diameter and distribution of PEK-C fibers.

Samples	Average Diameter (μm)	Standard Deviation of Diameter (μm)	Coefficient of Variation for Diameter (%)	Diameter Distribution Range (μm)
As-spun fibers	90.71	8.00	8.82	86–94
PO-21 rpm	27.23	3.76	13.80	21–35
PO-24 rpm	30.43	4.95	16.27	22–40

**Table 7:** Tensile properties of PEK-C fiber at different draw ratios.

Sample	Tensile Strength (cN/dtex)	Breaking Strength (cN/dtex)	Elongation at Break (%)
DR-2.5	0.560 ± 0.039	1.059 ± 0.089	13.02 ± 2.90
DR-2.0	0.526 ± 0.071	0.969 ± 0.086	41.33 ± 6.59
DR-1.5	0.499 ± 0.065	0.643 ± 0.041	80.79 ± 1.56
As-spun fibers	0.367 ± 0.089	0.490 ± 0.064	31.87 ± 3.02

Data are presented as mean ± standard deviation (SD), n = 5.

**Table 8:** Molecular chain orientation of PEK-C fibers at different draw ratios.

Sample	As-Spun Fibers	DR-1.5	DR-2.0	DR-2.5
Δn	—	0.07	0.08	0.09

## Data Availability

The original contributions presented in this study are included in this article. Further inquiries can be directed at the corresponding authors.
